# Supplementary material for: Prebiotic supplementation effect on Escherichia coli and Salmonella species associated with experimentally induced intestinal coccidiosis in rabbits
Source: PeerJ. 2021 Jan 22;9:e10714. doi: 10.7717/peerj.10714 (PMC7831364; doi:10.7717/peerj.10714)
Supplement: Supplemental Information 2 [file peerj-09-10714-s002.docx]

Figure showed the start of oocysts shedding and its peak.
