# Supplementary material for: Prebiotic supplementation effect on Escherichia coli and Salmonella species associated with experimentally induced intestinal coccidiosis in rabbits
Source: PeerJ. 2021 Jan 22;9:e10714. doi: 10.7717/peerj.10714 (PMC7831364; doi:10.7717/peerj.10714)
Supplement: Supplemental Information 3 [file peerj-09-10714-s003.docx]

S Table (1). The interpretation of Enterobacteriacae sensitivity test according to (CLSI/NCCLS, 2009)

| Antimicrobial  Discs | Disc  Potency  Mg/disc | Interpretation | | |
| --- | --- | --- | --- | --- |
|  |  | Zone diameter (mm) | | |
|  |  | Sensitive  ≤ | Intermediate | Resistant  ≥ |
| Amoxicillin + Clavulinic acid | 20-10µg | 18 | 14-17 | 13 |
| Chloramphenicol | 30µg | 18 | 13-17 | 12 |
| Ciprofloxacin | 5µg | 21 | 16-20 | 15 |
| Gentamicin | 10µg | 15 | 13-14 | 12 |
| Nalidixic acid | 30µg | 19 | 14-18 | 13 |
| Nitrofurantoin | 300 µg | 17 | 15-16 | 14 |
| Norfloxacin | 10 µg | 17 | 13-16 | 12 |
| Streptomycin | 10 µg | 15 | 12-14 | 11 |
| Trimethoprim-sulfamethoxazole | 1.25-23.75µg | 16 | 11-15 | 10 |
| Tetracycline | 30 µg | 15 | 12-14 | 11 |

S Table 2. Antimicrobial sensitivity tests of *E. coli* serotypes recovered from intestinal tissues of different groups

| No. | O.  Groups | Group | Antimicrobial agents | | | | | | | | | | | |
| --- | --- | --- | --- | --- | --- | --- | --- | --- | --- | --- | --- | --- | --- | --- |
|  |  |  | TE | SMZ | NA | SXT | GN | LEV | FFC | AML | UB | CiP | AK | N |
| 1 | O78 | PC | R | R | R | R | I | R | R | I | R | I | S | R |
| 2 | O78 | PC | R | R | R | I | I | R | R | I | R | S | I | R |
| 3 | O78 | PS | I | I | R | R | R | I | R | R | R | I | S | R |
| 4 | O125 | PC | R | I | R | R | R | R | R | S | R | S | S | R |
| 5 | O125 | PC | R | R | I | R | I | R | R | I | R | I | S | R |
| 6 | O152 | NC | R | R | R | R | R | R | R | R | R | R | S | R |
| 7 | O152 | NC | R | R | R | R | R | R | R | R | R | I | I | R |
| 8 | O152 | PC | I | R | R | R | R | R | R | I | R | R | I | R |
| 9 | O115 | PS | R | R | I | I | S | I | R | I | S | I | R | R |
| 10 | O168 | PC | R | R | R | R | I | R | R | I | R | R | S | R |
| 11 | O168 | NC | R | I | R | R | R | I | R | R | I | R | I | R |

NC= Negative control (non-prebiotic supplemented non-infected control

PC= Positive infected non-supplemented control

PS= Prebiotic supplemented infected group

S Table 3. Antibiotic sensitivity tests of *Salmonella* serovars

| *Salmonella*  Serovars | Group | Antimicrobial agents | | | | | | | | | | | |
| --- | --- | --- | --- | --- | --- | --- | --- | --- | --- | --- | --- | --- | --- |
|  |  | TE | SMZ | NA | SXT | GN | LEV | FFC | AML | UB | CiP | AK | N |
| *S.Macclesfield* | PC | R | R | I | I | I | S | I | I | R | S | S | I |
| *S.Canada* | NC | I | R | I | S | S | S | R | R | R | S | I | R |
| *S.Kisangani* | PC | R | I | R | S | S | I | R | R | I | S | R | I |
| *S.Kisangani* | PC | I | R | R | I | R | S | I | R | R | I | S | I |

NC= Negative control (non-prebiotic supplemented non-infected control

PC= Positive infected non-supplemented control
